# Community screening for iron deficiency in reproductive aged women: Lessons learnt from Australia

**DOI:** 10.1111/vox.13750

**Published:** 2024-10-13

**Authors:** Beth MacLean, Jayne Lim, Jess Fuller, Riki Wylie, Judie Yeleen Joo, Annas Al‐Sharea, Jaahnavi Cheyyur, Henry Ng, Sijing Zhang, Mubashshira Ahmed, Cory Dugan, Toby Richards

**Affiliations:** ^1^ School of Medicine University of Western Australia Perth Australia; ^2^ School of Health, Sport and Bioscience University of East London London UK

**Keywords:** anaemia, iron deficiency, women's health

## Abstract

**Background and Objectives:**

Reproductive‐aged women are at an increased risk of developing iron deficiency (ID). We aimed to develop a non‐invasive screening tool to identify ID in women and assess the acceptability of screening.

**Study Design and Methods:**

We screened women (age 18–49 years) in the community of Western Australia. Primary outcome: acceptability of screening, assessed by the feasibility of recruiting the required sample size (*n* = 323). Secondary outcomes: Hand grip strength, finger prick haemoglobin concentration (Hb), prevalence of heavy menstrual bleeding (HMB), diet, pregnancy history, blood donation, symptoms of ID and history of ID or anaemia (Hb < 120 g/L). Those with Hb <130 g/L and no history of iron therapy in the past 2 years were given referrals for venous full blood count and ferritin sampling.

**Results:**

Across 5 days, we recruited 640 eligible women. Of which, 178 (28%) had HMB and 79 (12%) were anaemic. Mean age was 33.5 ± 9.2 years, and mean Hb was 132.4 ± 11.9 g/L. In the past 2 years: 335 (52%) were diagnosed with ID or anaemia; 322 (50%) had taken oral iron; and 210 (33%) had an intravenous iron infusion. Vegetarian diets were followed by 89 (14%); 40 (6%) were regular blood donors; 290 (45%) had a previous pregnancy.

HMB increased the risk of symptoms of ID and having prior ID/anaemia diagnosis (67% vs. 47%) or treatment (*p* < 0.022). Hand grip strength showed a positive relationship with both Hb (adjusted *R* ^2^ = 0.012, *p* = 0.004) and ferritin (adjusted *R* ^2^ = 0.135, *p* = 0.005).

**Conclusion:**

ID screening was well accepted by women in the community, with high recruitment rates over a short period. Future screening tool development may consider incorporating hand grip strength and HMB assessment.

**Highlights**

- Iron deficiency (ID) screening was well accepted by reproductive‐aged women in the community.
- Women with heavy menstrual bleeding were more likely to report symptoms of ID and have a history of anaemia diagnosis.
- Hand grip strength showed a positive relationship with haemoglobin concentration and serum ferritin level.

## INTRODUCTION

During the reproductive years, women are at an increased risk of developing an iron deficiency (ID) [1]. During a typical flow period, women lose around 40–50 mL of blood, equating to 25 mg of iron [2]. Between 25% and 53% of women are thought to experience heavy menstrual bleeding (HMB) [2, 3], which the National Institute for Health and Care Excellence (NICE) defines as ‘excessive menstrual blood loss which interferes with a woman's physical, social‐emotional, and/or material quality of life’ [4]. Those with HMB are at an increased risk of developing an ID due to the greater quantity of iron lost through menstruation, which can be in excess of 40 mg per cycle [2].

Another common cause of ID during a woman's reproductive years is pregnancy. Each pregnancy increases the iron demand to expand the maternal blood volume and to supply both foetal and placental development. Typically, a 9‐month gestation period consumes 1200 mg of maternal iron, with a further 200 mg lost during labour and an additional 200 mg lost in the post‐partum period if the mother chooses to breastfeed [5]. This results in the loss of around half of a woman's bodily iron stores; thus, each sequential pregnancy puts a woman at an exponentially greater risk of becoming iron deficient.

Recent evidence from the US‐based National Health and Nutrition Examination Survey (NHANES) found that 38.6% of non‐pregnant women between the age of 12–21 years old had a ferritin <25 μg/L [6]. When following the consensus that a ferritin <50 μg/L may be early indicator of ID development [7], 77.5% of women from this US cohort had a ferritin <50 μg/L [6]. Despite this distinct prevalence, there are no formal screening guidelines targeting ID identification for reproductive‐aged women [1]. Having previously formed a case for implementing a corresponding screening programme [1], alongside developing a screening algorithm, we wanted to develop a corresponding screening tool and assess its acceptability within the population.

Iron is involved across many major bodily systems, and as such, a deficiency can present in a wide range of potential non‐specific symptoms, making diagnosis without venous blood testing challenging. As implementing routine blood testing is a costly approach to screening, we aimed to develop a non‐invasive, low‐cost screening tool that could be feasibly conducted in the community prior to confirmatory blood testing.

## MATERIALS AND METHODS

Between October 2023 and February 2024, five all‐day data collection events were conducted in a shopping centre in Perth Western Australia. Women between the age of 18–49 years were recruited to complete a self‐report questionnaire on risk factors for ID (Appendix 1 in Supporting Information). This questionnaire has been informed through a series of observational studies conducted by the study team previously, where common symptoms of ID and common risk factors for ID development have been identified [8, 9, 10].

The primary outcome of the current study was the acceptability of screening, assessed by the feasibility of reaching the study sample size. Secondary outcomes assessed in the questionnaire included relevant history on diet, pregnancy, blood donation, symptoms of iron deficiency, any prior diagnosis or treatment for ID or anaemia and prevalence of HMB in the cohort. The four‐item screener, developed by Fraser et al. [11], was used to identify HMB, where answering ‘yes’ to experiencing 2 or more of the following questions during a menstrual period was suggestive of HMB:

1. Flooding through clothes or bedding.
2. Need of frequent changes of sanitary towels or tampons (meaning changes every 2 h or less or 12 sanitary items per period).
3. Need of double sanitary protection (tampons and towels).
4. Pass large blood clots.

Haemoglobin concentration (Hb) was reported by finger prick testing (Hemocue Hb801). Muscle function was measured using a handgrip dynamometer (Jamar Hydraulic Hand Dynamometer) with the best of three grip strength attempts recorded on per hand and the greatest result of both hands deemed as the maximum grip strength.

For those with Hb < 130 g/L and no history of iron therapy in the past 2 years, outpatient referrals were given for venous testing, which reported full blood count (through spectrophotometry) and standard serum ferritin testing. Though anaemia will be defined as Hb < 120 g/L, a threshold of Hb < 130 g/L was used to account for variations in point‐of‐care‐testing [12]. Those with ferritin <30 μg/L were classed as iron deficient, and the data were used for a sub‐analysis to explore predictors of ID from the questionnaire. All participants provided informed consent and participant privacy was protected through secure encryption of the data and de‐identified analysis and reporting. Ethical approval was granted by the Human Research Ethics Office at the University of Western Australia (2022/ET000913).

### Sample size

The sample size was calculated using a confidence level of 95% with a *Z* score of 1.96 and margin of error of 5%. The population size of women between the age of 18–49 years in Western Australia was estimated to be 1.076 million according to the Australian Bureau of Statistics 2022 [13]. With HMB estimated to be reported in 30% of women during their reproductive years [2], the minimum sample size for the primary outcome would be *n* = 323. Anaemia is estimate to affect 29.9% of women during their reproductive years [14], therefore, a minimum sample size of *n* = 322 would be required to address this secondary endpoint.

### Statistical analysis

Complete case analysis was used, whereby only complete questionnaires were included and analysis was conducted in R (version 4.4.1). Continuous data were reported as mean ± standard deviation and analysed using *t* tests with Bonferroni correction testing. Categorical data were reported as number of responders with the percentage of responders within each group and analysed using Pearson's chi‐square test followed by Bonferroni correction testing. The relationship between variables was explored through experimental linear and logistic regression analysis, with non‐normally distributed data, as reported by Shapiro–Wilk testing, transformed logarithmically. Subgroup analysis was conducted by HMB status, anaemia status and ID status. Accuracy of finger prick Hb testing was assessed by plotting Hb values from those with venous samples on a calibration curve against their corresponding finger prick Hb result and assessing the confidence interval using 1000 bootstrap replicates with a 95% confidence interval.

## RESULTS

Over 5 days, we recruited 745 participants for screening, 105 of which were excluded due to either not being eligible for the study (not female or not 18–49 years old) or for submitting incomplete questionnaires. In total, we obtained 640 eligible datasets. The mean age of the cohort was 33.5 ± 9.2 years with a mean body mass index (BMI) of 24.5 ± 5.3 kg/m^2^ (Table 1). The mean Hb was 132.4 ± 11.9 g/L with 79 women being anaemic (12%). In the past 2 years, 335 (52%) had a diagnosis of ID or anaemia; 322 (50%) had taken oral iron and 210 (33%) had an intravenous iron infusion. Vegetarian diets were followed by 89 (14%); 40 (6%) were regular blood donors; 290 (45%) had a previous pregnancy.

**TABLE 1:** Demographics by heavy menstrual bleeding status.

	Non‐HMB (*n* = 462)	HMB (*n* = 178)	Total (*n* = 640)	Significance
Age	34.4 (±8.9)	31.1 (±9.5)	33.5 (±9.2)	**<0.001**
Hb (g/L)	132. 6 (±11.8)	132.0 (±12.2)	132.4 (±11.9)	1.000
Anaemia	51 (11%)	28 (16%)	79 (12%)	0.138
Height (cm)	163.8 (±7.8)	165.2 (±7.2)	164.2 (±7.6)	0.341
Weight (kg)	65.7 (±15.9)	67.7 (±14.3)	66.2 (±15.5)	1.000
BMI	24.4 (±5.5)	24.7 (±4.8)	24.5 (±5.3)	1.000
History of ID				
ID or anaemia in past 2 years	216 (47%)	119 (67%)	335 (52%)	**<0.001**
Taken oral iron in past 2 years	219 (47%)	103 (58%)	322 (50%)	**0.022**
Had an iron infusion	133 (29%)	77 (43%)	210 (33%)	**<0.001**
Had iron infusion in past 2 years	84 (18%)	50 (28%)	134 (21%)	**0.008**
Menstruation				
Periods in past 12 months	10.0 (±3.7)	10.2 (±3.2)	10.1 (±3.6)	1.000
Pregnancy				
History of pregnancy	226 (49%)	64 (36%)	290 (45%)	**0.004**
ID/anaemia during pregnancy	91 (20%)	37 (21%)	128 (20%)	**0.019**
Depression during pregnancy	41 (9%)	13 (7%)	54 (8%)	0.071
Hair loss during pregnancy	91 (20%)	36 (20%)	127 (20%)	**0.025**
Other risk factors				
Vegetarian	58 (13%)	31 (17%)	89 (14%)	0.143
Blood donor (past 2 years)	33 (7%)	7 (4%)	40 (6%)	0.186

*Note*: The table displays the cohort demographics with the non‐heavy menstrual bleeding (HMB) and HMB groups determined by the result of the four‐item screener developed by Fraser et al. [11]. Continuous data are displayed as mean ± standard deviation and analysed by *t*‐test. Prevalence data are reported as number of responders and percentage (%) of responders within the group. Significance values in bold indicate statistical significance (*p* < 0.05).

Abbreviations: BMI, body mass index; Hb, haemoglobin concentration; ID, iron deficiency.

Of the 640 women, 178 reported HMB (28%, Table 1); notably, HMB was more commonly reported in the younger cohort (31.1 ± 9.5 years vs. 34.4 ± 8.9 years, *p* < 0.001). Upon finger prick testing, those with HMB had a similar Hb to those without (132.0 ± 12.2 g/L vs. 132.6 ± 11.8 g/L, *p* = 1.000) and were no more likely to be classified as anaemic (16% vs. 11%, *p* = 0.138). However, those with HMB were more likely to report a history of ID or anaemia in the past 2 years (67% vs. 47%, *p* < 0.001) and were more likely to have received iron therapy (iron infusion: 43% vs. 29%, *p* < 0.001; oral iron: 58% vs. 47%, *p* = 0.022). Aside from chest pain, all listed symptoms of ID were significantly more frequently reported in those with HMB compared with those without (Table 2), and experimental logistic regression modelling with the symptoms in Table 2 found significant associations between shortness of breath, light‐headedness and brain fog with predicting HMB (*p* < 0.03).

**TABLE 2:** Symptoms of iron deficiency by heavy menstrual bleeding status.

	Non‐HMB (*n* = 462)	HMB (*n* = 178)	Total (*n* = 640)	Significance
Fatigue	323 (70%)	151 (85%)	474 (74%)	**<0.001**
Dizziness	208 (45%)	117 (66%)	325 (51%)	**<0.001**
Brain fog	161 (35%)	107 (60%)	268 (42%)	**<0.001**
Anxiety	167 (36%)	93 (52%)	260 (41%)	**<0.001**
Muscle weakness	132 (29%)	71 (40%)	203 (32%)	**0.008**
Shortness of Breath	93 (20%)	71 (40%)	164 (26%)	**<0.001**
Heart palpitations	69 (15%)	49 (28%)	118 (18%)	**<0.001**
Headaches	171 (37%)	103 (58%)	274 (43%)	**<0.001**
Hair loss	110 (24%)	59 (33%)	169 (26%)	**0.021**
Restless legs	71 (15%)	50 (28%)	121 (19%)	**<0.001**
Depression	57 (12%)	43 (24%)	100 (16%)	**<0.001**
Feeling cold	107 (23%)	68 (38%)	175 (27%)	**<0.001**
Exhaustion	93 (20%)	63 (35%)	156 (24%)	**<0.001**
Irritability	109 (24%)	80 (45%)	189 (30%)	**<0.001**
Shakiness	46 (10%)	36 (20%)	82 (13%)	**<0.001**
Pica	8 (2%)	9 (5%)	17 (3%)	**0.039**
Chest pain	28 (6%)	17 (10%)	45 (7%)	0.169
Fast heart rate	36 (8%)	31 (17%)	67 (10%)	**<0.001**
Bruising	66 (14%)	49 (28%)	115 (18%)	**<0.001**
Light‐headed	107 (23%)	85 (48%)	192 (30%)	**<0.001**
Vision problems	45 (10%)	37 (21%)	82 (13%)	**<0.001**
Tingling	37 (8%)	32 (18%)	69 (11%)	**<0.001**
Brittle nails	57 (12%)	45 (25%)	102 (16%)	**<0.001**
Dry skin	120 (26%)	70 (39%)	190 (30%)	**0.001**
Muscle soreness	114 (25%)	62 (35%)	176 (28%)	**0.013**
Joint pain	72 (16%)	50 (28%)	122 (19%)	**<0.001**
No symptoms	41 (9%)	2 (1%)	43 (7%)	**<0.001**

*Note*: The table displays the number of responders in each group (non‐heavy menstrual bleeding [HMB] vs. HMB) to symptoms commonly reported in iron deficiency. Prevalence data are reported as number of responders and percentage (%) of responders within the group. Significance values in bold indicate statistical significance (*p* < 0.05).

**FIGURE 1:**
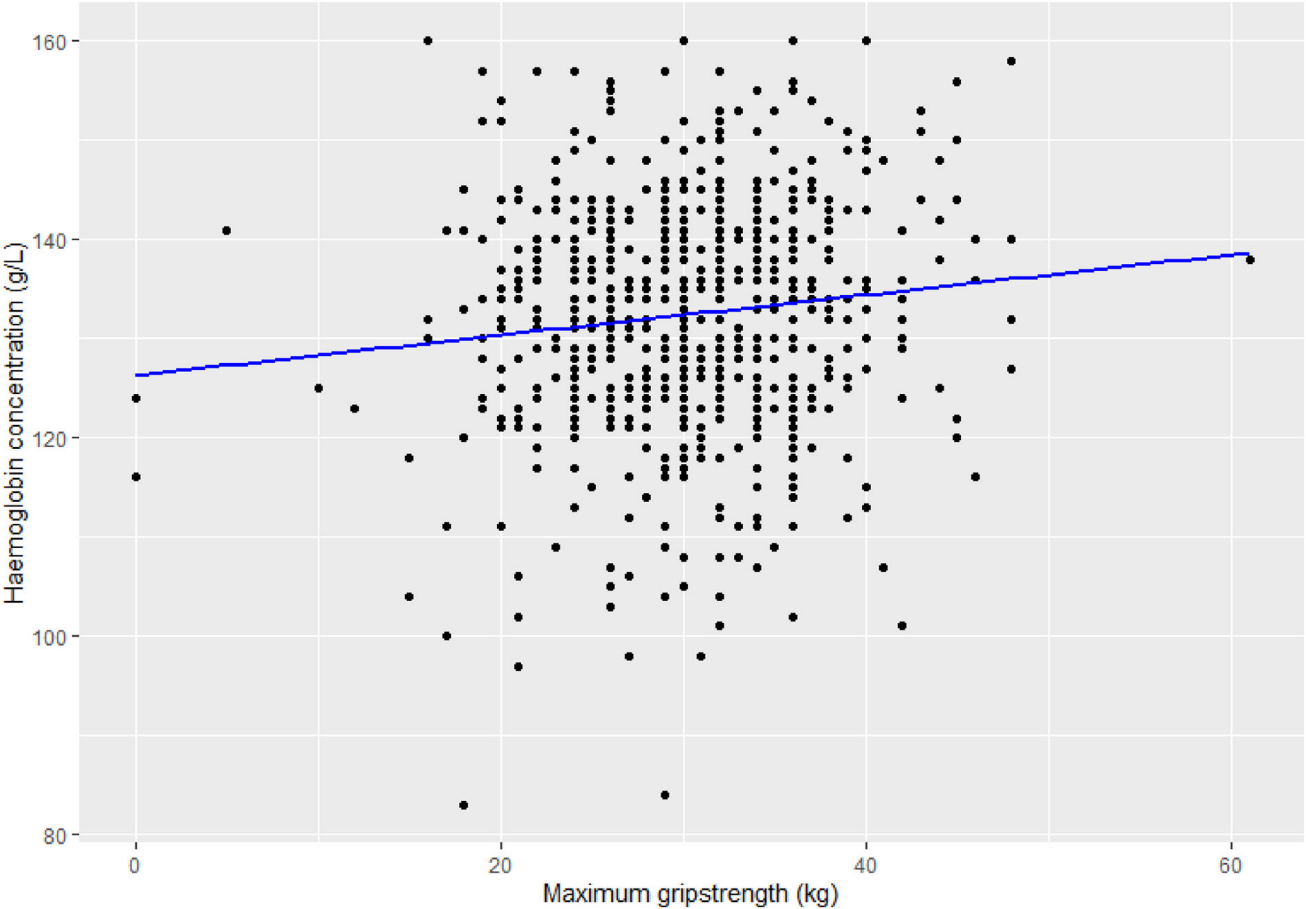
Linear regression analysis of the impact of haemoglobin concentration (Hb) on grip strength. The maximum grip strength of either hand recording is plotted against Hb for the entire cohort. The linear regression showed adjusted *R* ^2^ = 0.012, F‐statistic = 8.524, *p* = 0.004.

Neither HMB nor anaemia status impacted maximum grip strength (*p* > 0.05, Table 3); however, linear regression analysis of Hb against maximum grip strength showed a positive relationship (adjusted *R* ^2^ = 0.012, F‐statistic = 8.524, *p* = 0.004, Figure 1), although the low adjusted *R* ^2^ value suggests a weak relationship.

**TABLE 3:** Analysis of grip strength data by heavy menstrual bleeding status.

	Non‐HMB (*n* = 462)	HMB (*n* = 178)	Total (*n* = 640)	Significance
Right‐handed	419 (91%)	166 (93%)	585 (91%)	0.420
Right grip strength (kg)	29.3 (±6.7)	30.5 (±6.5)	29.7 (±6.7)	0.403
Left grip strength (kg)	27.1 (±6.5)	27.7 (±6.0)	27.2 (±6.4)	1.000
Maximum grip strength (kg)	29.9 (±6.7)	30.8 (±6.8)	30.1 (±6.7)	1.000
Sarcopenia (kg <16)	5 (1%)	1 (1%)	6 (1%)	0.877
Sarcopenia (kg <20)	20 (4%)	7 (4%)	27 (4%)	0.997

*Note*: The data report the distribution of right‐hand dominant participants and the maximum grip strength of three tests for both the left and right hand. The row titled ‘maximum grip strength’ then refers to the maximum score achieved regardless of hand. Sarcopenia responders were determined as those with a maximum grip strength <16 kg, in accordance with recommended guidelines for diagnosing sarcopenia in women from the European Working Group on Sacropenia in Older People (EWGSOP). Current consensus suggests revision to <20 kg for women, hence a secondary diagnostic range is displayed.

Abbreviation: HMB, heavy menstrual bleeding.

**FIGURE 2:**
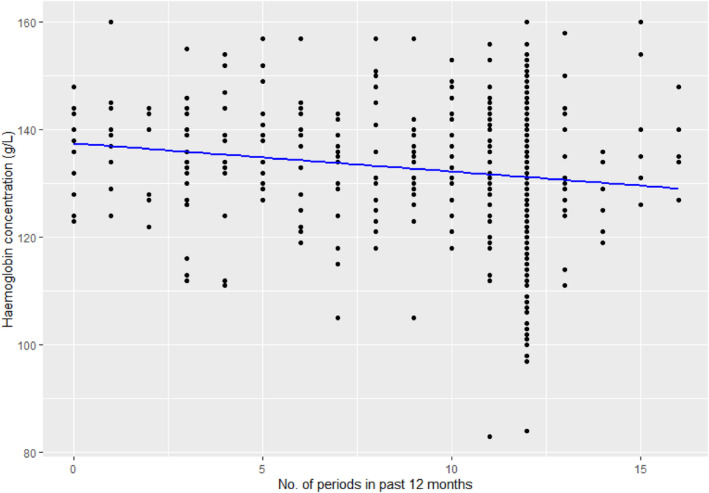
The influence of number of periods in a year on haemoglobin concentration (Hb). The number of periods reported in the past 12 months is plotted individually against Hb for the entire cohort. Linear regression analysis showed adjusted *R* ^2^ = 0.022, F‐statistic = 13.77 and *p* < 0.001.

When sub‐analysed by anaemia status, more menstrual periods were reported annually in those with anaemia when compared with the non‐anaemic women (11.0 ± 2.5 vs. 9.9 ± 3.7, *p* = 0.001). Similarly, linear regression analysis suggested a weak relationship where those with more periods in a year may be more likely to have a lower Hb (adjusted *R* ^2^ = 0.022, F‐statistic = 13.77 and *p* < 0.001, Figure 2).

Multivariate logistic regression analysis was conducted to explore the effects of HMB, vegetarian status, blood donation status and diet on anaemia status; however, no outcomes appeared to be statistically significant predictors of anaemia, with similar results when repeated as a linear regression model using the factors to predict Hb (*p* > 0.05). However, significant effects were observed in a linear regression model predicting Hb from reporting of symptoms of shortness of breath (*p* = 0.005), hair loss (*p* = 0.012), depression (*p* = 0.004) and brittle nails (*p* = 0.002). However, overall, a linear regression model utilizing the ID symptoms listed in Table 2 only explained 7.3% of variance in Hb (adjusted *R* ^2^ = 0.032, F‐statistic = 1.776, *p* = 0.010).

### Venous sample sub analysis

**FIGURE 3:**
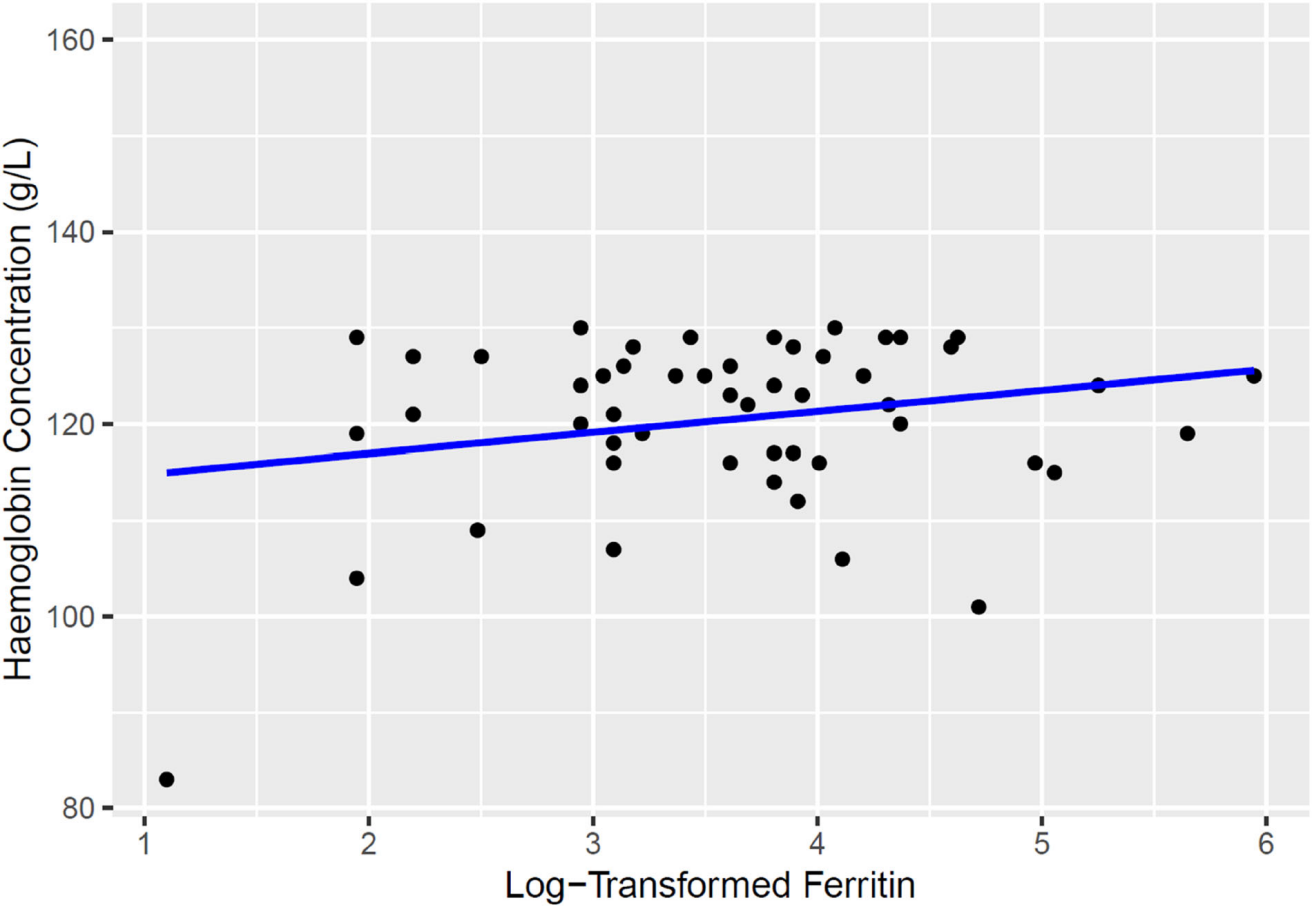
Venous haemoglobin concentration against log‐transformed ferritin. Linear regression analysis showed adjusted *R* ^2^ = 0.038, F‐statistic = 2.973, *p* = 0.091.

Of 150 referrals given to those with Hb < 130 g/L with no history of iron therapy in the past 2 years, 51 women went on for further venous blood sampling. Of 51 women, ID (ferritin <30 μg/L) was detected in 20 women (39%). Those with ID showed similar Hb values to those without (118.9 ± 11.1 g/L vs. 121.5 ± 7.1 g/L, *p* = 0.364) and were no more likely to be anaemic than those without an ID (40% vs. 35%, *p* = 1.000). Linear regression analysis of venous Hb against log‐transformed ferritin found a weak relationship between the two variables (adjusted *R* ^2^ = 0.038, F‐statistic = 2.973, *p* = 0.091, Figure 3).

Comparing those with ID to those without, there was no difference in ID/anaemia history in reporting of ID diagnosis or treatment in the past 2 years (*p* > 0.05) and no difference in ID risk factors (vegetarian, blood donor, pregnancy, HMB, period frequency: *p* > 0.05). ID symptoms were similarly reported between those with and without ID (*p* > 0.05, Table 4). However, linear regression analysis found significant effects on log‐transformed ferritin from the reporting of dizziness (*p* = 0.021), muscle weakness (*p* = 0.035), heart palpitations (0.015), irritability (*p* = 0.015) and muscle soreness (*p* = 0.008), while a model with all symptoms in Table 2 explained 63.2% of the variance in log‐transformed ferritin, though the predictors as a group did not significantly explain the variance (adjusted *R* ^2^ = 0.200, F‐statistic 1.464, *p* = 0.178). A logistic regression analysis was performed to assess the impact of HMB, blood donation status, vegetarian status and anaemia on the binary outcome variable ID, but no variable showed a statistically significant effect (*p* > 0.05), while similarly linear regression analysis to predict log‐transformed ferritin showed no effects (*p* = 0.774, adjusted *R* ^2^ = −0.039).

**TABLE 4:** Symptoms of iron deficiency by iron status.

	Non‐ID (*n* = 31)	ID (*n* = 20)	Total (*n* = 51)	Significance
Fatigue	25 (81%)	14 (70%)	39 (76%)	0.591
Dizziness	18 (58%)	11 (55%)	29 (57%)	1.000
Brain fog	11 (35%)	9 (45%)	20 (39%)	0.700
Anxiety	11 (35%)	7 (35%)	18 (35%)	1.000
Muscle weakness	6 (19%)	7 (35%)	13 (25%)	0.356
Shortness of breath	6 (19%)	6 (30%)	12 (24%)	0.591
Heart palpitations	7 (23%)	1 (5%)	8 (16%)	0.197
Headaches	9 (29%)	7 (35%)	16 (31%)	0.889
Hair loss	5 (16%)	7 (35%)	12 (24%)	0.225
Restless legs	5 (16%)	3 (15%)	8 (16%)	1.000
Depression	4 (13%)	2 (10%)	6 (12%)	1.000
Feeling cold	7 (23%)	3 (15%)	10 (20%)	0.761
Exhaustion	8 (26%)	5 (25%)	13 (25%)	1.000
Irritability	13 (42%)	6 (30%)	19 (37%)	0.573
Shakiness	3 (10%)	4 (20%)	7 (14%)	0.529
Pica	1 (3%)	0 (0%)	1 (2%)	1.000
Chest pain	4 (13%)	0 (0%)	4 (8%)	0.254
Fast heart rate	1 (3%)	1 (5%)	2 (4%)	1.000
Bruising	5 (16%)	2 (10%)	7 (14%)	0.838
Light‐headed	10 (32%)	2 (10%)	12 (24%)	0.136
Vision problems	4 (13%)	2 (10%)	6 (12%)	1.000
Tingling	1 (3%)	1 (5%)	2 (4%)	1.000
Brittle nails	4 (13%)	2 (10%)	6 (12%)	1.000
Dry skin	8 (26%)	5 (25%)	13 (25%)	1.000
Muscle soreness	4 (13%)	7 (35%)	11 (22%)	0.127
Joint pain	6 (19%)	3 (15%)	9 (18%)	0.982
No symptoms	2 (6%)	1 (5%)	3 (6%)	1.000

*Note*: The table displays the number of responders in each group (non‐iron deficient [ID] vs. ID) to symptoms commonly reported in iron deficiency. Prevalence data are reported as number of responders and percentage (%) of responders within the group.

**FIGURE 4:**
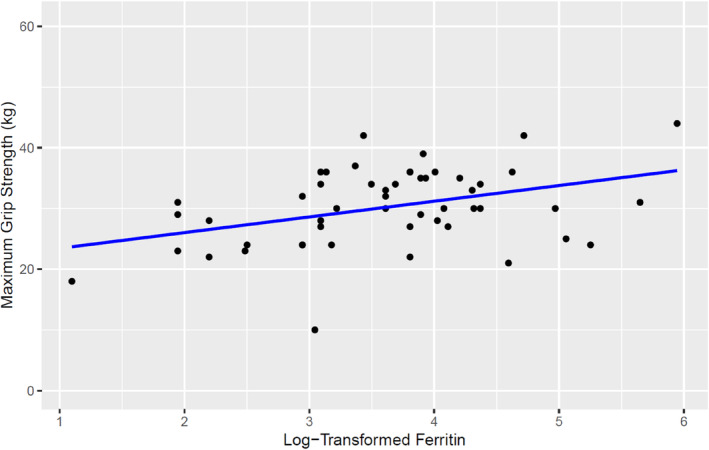
Relationship between grip strength and log‐transformed ferritin. Linear regression analysis showed adjusted *R* ^2^ = 0.135, F‐statistic = 8.829, *p* = 0.005.

Those with ID had a lower grip strength than those without (27.0 ± 6.6 vs. 32.3 ± 5.6, *p* = 0.006). Similarly, linear regression analysis showed a positive relationship between maximum grip strength and log‐transformed ferritin (adjusted *R* ^2^ = 0.135, F‐statistic = 8.829, *p* = 0.005, Figure 4).

**FIGURE 5:**
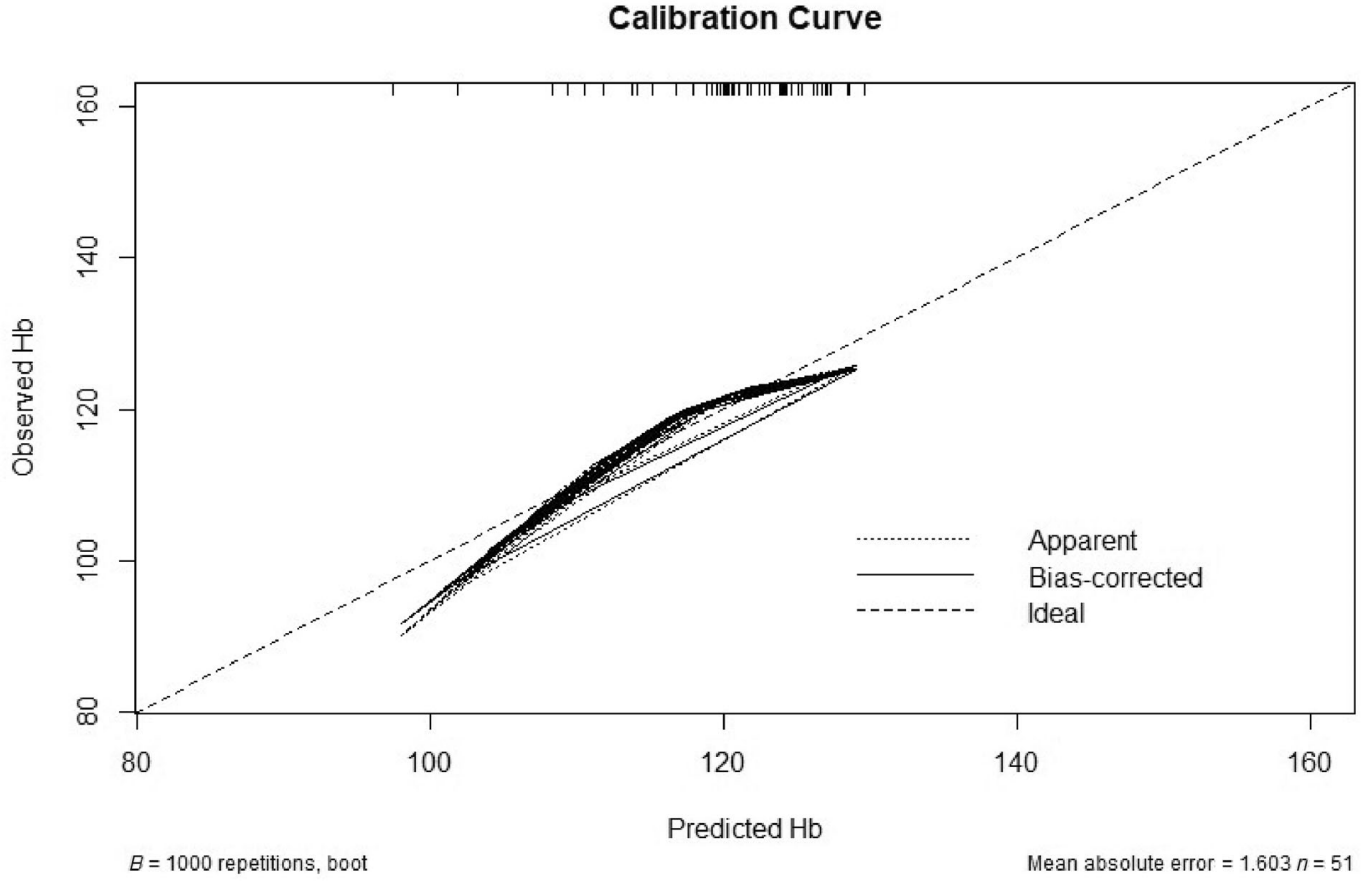
Accuracy of finger prick testing haemoglobin concentration (Hb). The above diagram displays the calibration curve of observed Hb (venous sampling) against predicted Hb (capillary/finger prick sampling). On 1000 bootstrap replicates with a 95% confidence interval, the accuracy of finger prick sampling lies between 4.941 and 7.696 g/L.

Finally, to assess accuracy of finger prick Hb testing, a calibration curve was created by plotting the finger prick Hb results against the corresponding venous Hb values and assessing the accuracy of Hb prediction using 1000 bootstrap replicates with a 95% confidence interval (Figure 5). The accuracy of finger prick Hb sampling was deemed to lie within ±8 g/L of the venous Hb sample.

## DISCUSSION

We observed that a self‐report questionnaire, hand grip strength and finger prick Hb testing was well accepted by women of reproductive age in the community of Western Australia for screening ID. The study was hosted as a series of five events in a shopping centre, however, due to the simplicity of recruitment and public interest, this methodology of screening could be successful strategy to implement in pharmacy and primary health care settings [1].

From the results of the study, we can observe that there is a substantial prevalence of HMB within the community of reproductive‐aged women in Perth, Western Australia. Affecting 28% of the cohort, the prevalence of HMB falls within a similar range to that observed in the United Kingdom of 25%–53% prevalence [2, 3]. Previous studies have found that HMB decreases aspects of a woman's quality of life [3, 15]; our study builds on this understanding through observation of the evident symptomatic burden associated with HMB. Although the symptoms explored in this study were non‐specific symptoms frequently associated with ID [2, 9, 10], they are widespread in systemic function and imply the extent of the burden HMB presents.

We also found in our experimental logistic and linear regression analysis, that various symptoms of ID have potential strength in predicting Hb and ferritin, although the latter was expected due to the nature of creating this list from frequently reported symptoms of ID in studies of women of reproductive age [2, 9, 10]. Incorporation of these associated symptoms should be considered in the development of an ID predictive algorithm in future work.

In line with observations in elderly iron‐deficient cohorts [16, 17], our results observed a positive relationship with hand grip strength for both ferritin and Hb. This supports the current understanding that iron is required for muscle function and that ID can negatively impact physical performance [18, 19]. Further exploration into pre‐ and post‐iron therapy muscle function would be beneficial to enhance our understanding of the relationship and the role of muscle function in identifying those at risk of an ID.

In terms of associative factors with ID, neither HMB nor anaemia status appeared to predict ID in the subgroup analysis. Referring patients with a Hb < 130 g/L provided an ID prediction rate of 39%. Based on prevalence estimates, we would expect a 40%–55% prevalence of ID in a random sample of women of reproductive age [20]; therefore, referral based on Hb thresholds may not be a useful means of screening for ID. This is likely due to the frequency of non‐anaemic ID in women of reproductive age [21], which is further supported by the lack of relationship between Hb and ferritin observed in this study. Notably, this analysis was limited by the low uptake of further blood testing and did not account for inflammation, which may have overlooked cases of functional ID. In addition, the lack of consensus regarding the definitions of both anaemia and ID in the otherwise healthy female population may be contributing factors [1].

In terms of further limitations, this study did not account for contraceptive use, which may influence differences in HMB prevalence and thus anaemia prevalence. Additionally, we were unable to account for recall bias; therefore, data pertaining to history of ID/anaemia diagnosis and previous treatment is liable to participant reporting error. Furthermore, the shopping centre setting was used to provide an easily accessible screening service located in a high‐traffic setting to enhance recruitment, thus we do appreciate that this setting may not be reflective of the general population and may have been of greater interest to those conscious of ID history.

In conclusion, our protocol for ID screening was well accepted by women in the community of Perth, Western Australia. There appears to be a relationship between ID, HMB and grip strength which should be considered for further development of a screening tool.

## CONFLICT OF INTEREST STATEMENT

The authors declare no conflicts of interest.

## Supporting information


**Data S1.** Supporting Information.

## Data Availability

The data that support the findings of this study are available on request from the corresponding author. The data are not publicly available due to privacy or ethical restrictions.

## References

[1] MacLean B , Sholzberg M , Weyand AC , Lim J , Tang G , Richards T . Identification of women and girls with iron deficiency in the reproductive years. Int J Gynecol Obstet. 2023;162:58–67. 10.1002/ijgo.14948 37538015

[2] Dugan C , MacLean B , Cabolis K , Abeysiri S , Khong A , Sajic M , et al. The misogyny of iron deficiency. Anaesthesia. 2021;76:56–62. 33682094 10.1111/anae.15432

[3] Karlsson TS , Marions LB , Edlund MG . Heavy menstrual bleeding significantly affects quality of life. Acta Obstet Gynecol Scand. 2014;93:52–57. 24266506 10.1111/aogs.12292

[4] National Institute for Health and Care Excellence . Heavy menstrual bleeding: assessment and management. NICE guideline [NG88]. National Institute for Health and Care Excellence; 2018. 34101395

[5] Breymann C , Honegger C , Hösli I , Surbek D . Diagnosis and treatment of iron‐deficiency anaemia in pregnancy and postpartum. Arch Gynecol Obstet. 2017;296:1229–1234. 28940095 10.1007/s00404-017-4526-2

[6] Weyand AC , Chaitoff A , Freed GL , Sholzberg M , Choi SW , McGann PT . Prevalence of iron deficiency and iron‐deficiency anemia in US females aged 12‐21 years, 2003‐2020. JAMA. 2023;329:2191–2193. 37367984 10.1001/jama.2023.8020 PMC10300696

[7] Tang GH , Sholzberg M . The definition of iron deficiency—an issue of health equity. JAMA Netw Open. 2024;7:e2413928‐e. 38848071 10.1001/jamanetworkopen.2024.13928

[8] Bruinvels G , Burden R , Brown N , Richards T , Pedlar C . The prevalence and impact of heavy menstrual bleeding (menorrhagia) in elite and non‐elite athletes. PLoS One. 2016;11:e0149881. 26901873 10.1371/journal.pone.0149881 PMC4763330

[9] Bruinvels G , Burden RJ , Cushway T , Brown N , Pedlar C , Richards T . The impact of heavy menstrual bleeding (menorrhagia) and iron status in exercising females. Br J Sports Med. 2017;51:304.

[10] Dugan C , Scott C , Abeysiri S , Baikady RR , Richards T . The need to screen for anemia in exercising women. Medicine (Baltimore). 2021;100:e27271. 34596123 10.1097/MD.0000000000027271 PMC8483825

[11] Fraser IS , Mansour D , Breymann C , Hoffman C , Mezzacasa A , Petraglia F . Prevalence of heavy menstrual bleeding and experiences of affected women in a European patient survey. Int J Gynecol Obstet. 2015;128:196–200. 10.1016/j.ijgo.2014.09.027 25627706

[12] Gehring H , Hornberger C , Dibbelt L , Roth‐Isigkeit A , Gerlach K , Schumacher J , et al. Accuracy of point‐of‐care‐testing (POCT) for determining hemoglobin concentrations. Acta Anaesthesiol Scand. 2002;46:980–986. 12190799 10.1034/j.1399-6576.2002.460809.x

[13] Australian Bureau of Statistics . Regional population by age and sex. ABS. Available from: https://www.abs.gov.au/statistics/people/population/regional-population-age-and-sex/latest-release. Last accessed 14 Apr 2024.

[14] World Health Organization . WHO global anaemia estimates, 2021 edition: global anaemia estimates in women of reproductive age, by pregnancy status, and in children aged 6–59 months. Available from: who.int/data/gho/data/themes/topics/anaemia_in_women_and_children. Last accessed 14 Apr 2024.

[15] Kocaoz S , Cirpan R , Degirmencioglu AZ . The prevalence and impacts heavy menstrual bleeding on anemia, fatigue and quality of life in women of reproductive age. Pak J Med Sci. 2019;35:365. 31086516 10.12669/pjms.35.2.644 PMC6500811

[16] Ho V , Lee C‐T , Merchant RA . The “iron tale”‐iron indices and handgrip strength in community‐dwelling adults. Aging Clin Exp Res. 2022;34:3025–3032. 36149625 10.1007/s40520-022-02242-5

[17] Hammer T , Braisch U , Rothenbacher D , Denkinger M , Dallmeier D . Relationship between hemoglobin and grip strength in older adults: the ActiFE study. Aging Clin Exp Res. 2024;36:59. 38451343 10.1007/s40520-024-02698-7 PMC10920471

[18] Beard JL . Iron biology in immune function, muscle metabolism and neuronal functioning. J Nutr. 2001;131:568S–580S. 11160590 10.1093/jn/131.2.568S

[19] Stugiewicz M , Tkaczyszyn M , Kasztura M , Banasiak W , Ponikowski P , Jankowska EA . The influence of iron deficiency on the functioning of skeletal muscles: experimental evidence and clinical implications. Eur J Heart Fail. 2016;18:762–773. 26800032 10.1002/ejhf.467

[20] Milman N , Taylor CL , Merkel J , Brannon PM . Iron status in pregnant women and women of reproductive age in Europe. Am J Clin Nutr. 2017;106:1655S–1662S. 29070543 10.3945/ajcn.117.156000 PMC5701710

[21] Tang GH , Sholzberg M . Iron deficiency anemia among women: an issue of health equity. Blood Rev. 2023;64:101159. 38042684 10.1016/j.blre.2023.101159

