# Supplementary material for: Community screening for iron deficiency in reproductive aged women: Lessons learnt from Australia
Source: Vox Sang. 2024 Oct 13;120(1):22–31. doi: 10.1111/vox.13750 (PMC11753822; doi:10.1111/vox.13750)
Supplement: Supplementary file 1 — Data S1. Supporting Information. [file VOX-120-22-s001.docx]

**Appendix 1**

CAPRI Questionnaire

Start of Block: Participant Information and Consent Form

Q1 Online Survey Participant Information

Q2 Are you a woman aged 18 - 49 years old?

- Yes
- No

Skip To: End of Survey If Are you a woman aged 18 - 49 years old? = No

Q3 I consent to participate in this research project

- Yes
- No

Skip To: End of Survey If I consent to participate in this research project = No

Q4 Please sign here

Q5 First name

________________________________________________________________

Q6 Last name

________________________________________________________________

Q7 Email address (for the purpose of communicating the results)

________________________________________________________________

| 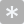 |
| --- |

Q8 Date of birth (dd/mm/yyyy)

________________________________________________________________

End of Block: Participant Information and Consent Form

Start of Block: Personal Details

| 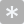 |
| --- |

Q9 What is your height? (cm)

________________________________________________________________

| 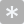 |
| --- |

Q10 What is your weight? (kg)

________________________________________________________________

End of Block: Personal Details

Start of Block: Mensuration

Q11 To your knowledge, have you ever had anaemia or iron deficiency in the past 2 years?

- Yes
- No

Q12 Have you taken oral iron tablets in the past 2 years?

- Yes
- No

Q13 Have you ever had an iron infusion?

- Yes
- No

Display This Question:

If Have you ever had an iron infusion? = Yes

| 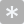 |
| --- |

Q14 How many years ago was your iron infusion?

________________________________________________________________

End of Block: Mensuration

Start of Block: Block 7

Q15 Approximately how many periods have you had in the last 12 months?

|  | 0 | 1 | 2 | 3 | 4 | 5 | 6 | 7 | 8 | 9 | 10 | 11 | 12 | 13 | 14 | 15 | 16 |
| --- | --- | --- | --- | --- | --- | --- | --- | --- | --- | --- | --- | --- | --- | --- | --- | --- | --- |

| Number of periods () | 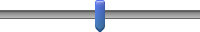 |
| --- | --- |

Q16 In regards to your period, have you ever experienced any of the following? (Please select all that apply)

- Flooding through clothes or bedding
- Need of frequent changes of sanitary towels or tampons (meaning changes every 2 hours or less, or 12 sanitary items per period).
- Need of double sanitary protection (tampons and towels)
- Pass large blood clots
- None of the above

End of Block: Block 7

Start of Block: Iron Deficiency Symptoms and Impact

Q17 Below are a list of symptoms- What are your symptoms of iron deficiency? (Please pick all that apply to you)

- Fatigue
- Dizziness
- Brain Fog
- Anxiety
- Muscle Weakness
- Shortness of Breath
- Heart Palpitations
- Headaches
- Hair Loss
- Restless Legs
- Depression
- Feeling Cold
- Exhaustion but difficulty falling asleep
- Irritability
- Shakiness
- Pica (craving ice, clay or other non-foods)
- Chest Pain
- Fast Heart Rate
- Bruising
- Lightheaded
- Vision Problems
- Tingling
- Brittle Nails
- Dry Skin
- Muscle Soreness
- Joint Pain
- No symptoms

End of Block: Iron Deficiency Symptoms and Impact

Start of Block: Block 6

Q18 Have you donated blood in the past 2 years?

- Yes
- No

Q19 Do you follow a vegetarian, vegan, pescatarian or similar diet?

- Yes
- No

End of Block: Block 6

Start of Block: Block 5

Q20 Have you ever had a pregnancy?

- Yes
- No

Display This Question:

If Have you ever had a pregnancy? = Yes

Q21 How many children do you have?

|  | 0 | 1 | 2 | 3 | 4 | 5 | 6 | 7 | 8 | 9 | 10 |
| --- | --- | --- | --- | --- | --- | --- | --- | --- | --- | --- | --- |

| Number of children () | 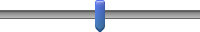 |
| --- | --- |

Display This Question:

If Have you ever had a pregnancy? = Yes

| 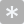 |
| --- |

Q22 How many years ago was your last child born?

________________________________________________________________

Display This Question:

If Have you ever had a pregnancy? = Yes

Q23 At your last childbirth, were you known to be iron deficient or anaemic?

- Yes
- No

Display This Question:

If Have you ever had a pregnancy? = Yes

Q24 At your last childbirth, did you suffer from hair loss?

- Yes
- No

Display This Question:

If Have you ever had a pregnancy? = Yes

Q25 At your last childbirth, did you suffer from postpartum depression?

- Yes
- No

End of Block: Block 5

Start of Block: Block 6

Q26 Please see the team for the handgrip strength test and anaemia test to answer the following questions

Q27 Dominant Hand

- Right
- Left

| 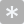 |
| --- |

Q28 Right hand grip

________________________________________________________________

| 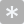 |
| --- |

Q29 Left hand grip

________________________________________________________________

| 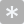 |
| --- |

Q30 Once you receive your haemoglobin concentration from the finger prick test, please input your result below (just the numbers no unit)

________________________________________________________________

End of Block: Block 6
